# Documentation as composing: how medical students and residents use writing to think and learn

**DOI:** 10.1007/s10459-022-10167-x

**Published:** 2022-11-01

**Authors:** Dillon Bowker, Jacqueline Torti, Mark Goldszmidt

**Affiliations:** 1 Schulich School of Medicine and Dentistry, Western University, London, Canada; 2 Present Address: Division of General Surgery, Department of Surgery, Dalhousie University, Halifax, Canada; 3 Department of Medicine, Schulich School of Medicine and Dentistry, London, Canada; 4 Faculty of Education, Western University, London, Canada; 5 Centre for Education Research and Innovation, Schulich School of Medicine and Dentistry, Western University, London, Canada; 6 Division of General Internal Medicine, Department of Medicine, Centre for Education Research and Innovatio, Western University, London, Canada; 7 London Health Sciences Centre (LHSC), 339 Windermere Road, London, ON N6A 5A5 Canada

**Keywords:** Clinical documentation, Clinical learning, Clinical reasoning, Composing, Internal medicine, Rhetorical genre theory, Writing

## Abstract

Some educators have described clinical documentation as “scut”. Research in medicine has focused on documentation’s communicative value and not its function in learning. With time being an important commodity and electronic health records changing how we document, understanding the learning value of documentation is essential. The purpose of this study was to explore how trainee composing practices shape learning. Qualitative methods employing Rhetorical Genre Theory were used to explore clinical documentation practices among medical trainees. Data collection and analysis occurred in iterative cycles. Data included field notes and field interviews from 110 h of observing junior trainees and senior internal medicine residents participating in patient admission and follow-up visits. Analysis was focused on Paré and Smart’s framework for studying documentation as composing. From a composing lens, documentation plays a vital role in learning in clinical settings. Junior trainees were observed to be reliant on using writing to support their thinking around patient care. Before patient encounters, writing helped trainees focus on what was already known and develop a preliminary understanding of the patient’s problem(s). After encounters, writing helped trainees synthesize the data and develop an assessment and plan. Before and after the encounter, through writing, trainees also identified knowledge and data collection gaps. Our findings highlight clinical documentation as more than a communication task. Rather, the writing process itself appeared to play a pivotal role in supporting thinking. While some have proposed strategies for reducing trainee involvement, we argue that writing can be time well spent.

## Introduction

In clinical learning settings, time is a precious commodity (Block et al., 2013; Chaiyachati et al., 2019). While direct patient care activities are typically viewed as time well spent for both learning and patient care, indirect care activities such as clinical documentation have been viewed as wasted time for trainees due to their low educational value (Dresselhaus et al., 1998; Petrany, 2013),(Sinsky et al., 2013; van Schaik, Reeves et al., 2019). Such activities may be relegated to the category of ‘scut’ (i.e., low educational value and menial work) (Dresselhaus et al., 1998; Petrany, 2013) with strategies implemented to free trainees from such tasks (Sinsky et al., 2013; van Schaik et al., 2019). However, the assessment of clinical documentation as “scut” is taking place in the absence of systematic exploration of its role in trainee education. Before we purge it from training environments, we should be certain of not only its drawbacks but also its affordances.

There are many types of clinical documents that trainees may be required to complete daily. While some are largely administrative (e.g., insurance forms), many are directly tied to patient care, such as admission notes, progress notes and discharge summaries, and each has its own specific requirements. These documentation types are a part of trainees’ day to day work across all levels of training, from their time as medical students up to senior residency. To date, clinical documentation research in medical education has largely focused on: problematizing the time spent on it, which at times conflates administrative documentation with other types of documentation (Petrany, 2013; Dresselhaus et al., 1998; Sinsky et al., 2013; van Schaik et al., 2019);learning how to do it (DeLeon et al., 2018; Opila, 1997; Rowlands et al., 2016); and the importance of doing it well (Cadieux & Goldszmidt, 2017; Goldszmidt et al., 2014). The potential role it plays in trainee learning and how it changes through their medical education, however, has not been studied. Whereas most of the literature conceptualizes writing as a mere mechanical task and thus focuses on its products, rhetorical genre theory (RGT) conceptualizes documentation as an important social activity, one that shapes and is shaped by its contexts of production and use. More specifically, it draws attention to typified forms of writing—genres—that are associated with recurrent situations and that serve particular goals (Bawarshi, 2001; Bawarshi & Reiff, 2010; Bazerman, 2009; Schryer, 2011). For example, the admission note is both a workplace and education genre. It serves clinical care purposes (Goldszmidt et al., 2014), of course, but its form also serves educational goals such as instructing medical students as to what and what not to attend to when seeing patients(Schryer, 2011). Moreover, it can also serve to help guide what and how they present their patient to the attending physician; if they did not collect the right information or they present in an unexpected sequence, they will likely be corrected and will reconsider what they collect, document and present for future patients (Goldszmidt et al., 2012). It may also support other forms of learning. In short, RGT suggests that clinical documentation may play a more significant role in supporting and guiding thinking than has previously been studied in medicine.

According to the RGT researchers Paré and Smart, the actual writing of a document (e.g. an admission note or progress note) is part of a larger task that they refer to as composing (Paré & Smart, 1994). In medicine, while much has been written about clinical reasoning and how we learn to reason (Pinnock et al., 2019; HG and S, 2015; Koufidis et al., 2020), the connections between composing and thinking (reasoning) have not been adequately explored. Composing involves the following activities: (1) initiating event (e.g. a new consult); (2) information gathering (e.g. the clinical encounter and reading of prior patient documents); (3) analysis of information; (4) individual writing and rewriting; (5) collaborative activities (e.g. reviewing with a more senior clinician) and; (6) the technology of production (e.g. writing, typing or dictating the clinical note) (Paré & Smart, 1994). Studies of these aspects of composing from an RGT perspective have helped shape teaching practices in various settings (Bawarshi & Reiff, 2010). Similar studies in medical contexts can yield insights into how trainees, in interacting with genres like the admission note, can learn (or fail to learn): how to read and analyze new and existing data; what the task expectations are and; how to prepare for the collaborative aspects of composing (i.e., case review).

To further our understanding of the learning value of writing during clinical work and to inform education practices around clinical documentation, the purpose of this study was to explore trainee composing practices in the context of a clinical setting where composing is used to perform clinical work.

## Method

This was a rhetorical genre study based on the research methodology proposed by Paré and Smart for studying composing, which is a theory-informed method for observing and exploring genre composition in real-world settings (Paré & Smart, 1994). The research team consisted of a pre-clinical medical student (DB) who was trained to participate in observational fieldwork by the senior author (MG)—an experienced qualitative researcher with expertise in both medicine and RGT—and a research associate (JT) who worked with MG and who is also experienced in qualitative observational research.

### Setting & sampling

Between June 2018 and August 2019, data were collected from six internal medicine inpatient teaching teams spread across two hospitals of an academic health sciences centre in Ontario, Canada.

Both purposeful and theoretical sampling were employed. Purposeful sampling was used at the trainee, location and shift levels. Trainees were sampled to capture a range of experience across and within years. Sampling from two different hospitals accounted for cultural and workplace practice variations, while observation of both daytime shifts and overnight call captured variations in workflow and team interactions. Later in data collection, we also engaged in theoretical sampling to help enrich and refine our emerging understanding of the phenomenon. For example, over time we recognized the need to focus more heavily on observations of on call shifts instead of daytime shifts. We were also able to incorporate questions into our field interviews to better interrogate aspects of our data that we had observed in previously but did not fully understand or to test our evolving theories. This iterative process thus allowed our data to adapt and properly inform our emerging understanding.

To minimize participant reactivity (Paradis & Sutkin, 2017), we conducted sustained observations and limited participants’ awareness of our specific research goal to further mitigate this concern. In obtaining consent, participants were informed about a more generic study goal of observing communication and documentation practices.

### Data collection

A medical student collected data as they were able to build rapport with trainees during extended observations, understand the medical context, and create a non-evaluative environment. Consistent with RGT informed research principles (Charmaz, 2014; Schryer, 2011), our data were collected through fieldnotes from direct observations and audio-recorded field interviews on the inpatient wards and in the emergency room.

A total of 26 observations were conducted. We observed six senior medical students (clinical clerks (CC)) rotating through their internal medicine core rotation, six junior internal medicine residents in their first post-graduate year (PGY1), one family medicine resident (PGY1) and eight senior internal medicine residents (3 PGY2s, 5 PGY3s). Eight field observations were conducted with junior trainees (medical students and PGY 1 residents) during daytime shifts on the wards. A further 18 observations were conducted during overnight call (junior trainees) and Emergency Department Consultation (senior trainees) shifts. During daytime shifts, trainees were observed during morning rounds, then shadowed as they saw assigned patients. During on-call shifts, trainees were observed at handover, then shadowed for several hours as they consulted on new admissions and managed overnight inpatient issues.

In both settings, the focus of observations was on the trainees’ multiple stages of composing. Observations occurred over more than 110 h, with one to four shifts observed for each participant. Documentation practices observed included both written notes (e.g., daily progress notes) and dictated notes (e.g., admission notes and discharge summaries). Field interviews accompanied observations – brief interviews of 1–10 min in duration performed, as time allowed, and at multiple time points throughout any given observation period. Using a *live scribe* pen and notebook (“8 GB Echo Smartpen—Livescribe Smartpen—Livescribe Inc. (US),” n.d.) so as to make recording unobtrusive, these interviews, when possible and with prior consent, were audio-recorded and subsequently transcribed by DB. Through these field interviews, the thinking behind practices was made visible. This approach is very similar to the one used by clinical reasoning researchers (Koufidis et al., 2020; Pinnock et al., 2019). Early in the process, the field interviews were used to explore observational findings. Over time, with repeated exposure and analysis of existing data, thinking could be largely inferred based on observations of how a particular task was carried out. For example, when trainees are observed to go back and forth between the medication list and the past medical history, it is clear that they are trying to reconcile which medications go with each of the patient’s chronic active problems. Similarly, there is a large difference between writing out the list of medications in any order versus re-configuring the list so that medications for each chronic active problem are kept together.

### Analysis

Principles of rigour, similar to those posited by constructivist grounded theory, were used to inform data collection and analysis, which took place in iterative cycles with the analysis of existing data informing subsequent data collection (Charmaz, 2014). Reflexive memos were used to maintain an understanding of the research team’s position relative to the participants. For example, DB, being a medical student, used memoing to grapple with his observations and ensuing judgements. Early on, these memos and reflections were used during team meetings to unpack some of the complexity of clinical practice that he was observing. This allowed him to uncouple the clinical practice and his own knowledge gaps from the documentation practices themselves, which were the focus of the study. We also employed constant comparison, analyzing earlier data for new themes that emerged later in the process.

For the initial analysis, thematic coding of three field notes was conducted independently by DB to generate a broad set of codes representing phenomena observed in the field in relation to composing practices.^16^ The initial coding was conducted manually, going line by line through the transcripts. These codes were then discussed with MG and JT. The initial set of codes was condensed and then focused coding was applied by DB to additional transcripts. A series of regular meetings—10 in total—were had between the research team to review and refine the coding process and develop an understanding of how composing is enacted by junior and senior trainees on the internal medicine service. Through the ongoing cycles of collection and analysis, the codes were refined and used to develop a theory of how trainees composing relates to learning. Theoretical sampling was also used to inform further sampling and identify and reconcile discrepant data.

## Results

Across the observations, we were able to identify, confirm and contextualize all of the composing processes described by Paré and Smart. The processes were identified across the data, including admission note writing by junior and senior trainees, ward progress notes written by junior trainees and ward notes written when junior trainees were called to assess a patient. For junior trainees, composing always included writing, as will be elaborated on below. For routine cases, senior residents could compose by assembling documents from the medical record and dictating from these without first creating a written template.

Regardless of training level, trainees appeared to use and described using composing to help frame their thinking about a patient. Depending on the individual trainee, however, more or less time appeared to be spent thinking; for some of the junior trainees, the task was approached in a more formulaic way, trying to add something, in writing, into every section of the note but without necessarily attempting to make sense of it all.

Two important periods were identified and self-evidently labelled *before and after the patient encounter*. Both periods contained a set of tasks that trainees used writing to help them address. Some were unique to one of the periods, while others were present in both. Additionally, we identified differences in how they approached tasks in the time periods before and after the patient encounter and how this changed with experience. The following sections will explore the tasks seen in both periods more thoroughly. Short representative quotations are used throughout to illustrate key findings. In these, we use the short form CC (clinical clerk) to represent medical students, IM to represent internal medicine residents and PGY1-3 to represent the post-graduate year of the resident being observed. The numbers after each abbreviation represent their random number generated ID. For those interested in more detailed data, the more elaborated quotations have been included in an online Appendix Tables 1, 2 and 3. The table, section and row where they are found are indicated at the end of the quotation (e.g., Table 1-1A).

### Before the patient encounter

Before all encounter types, composing was focused on understanding what is already known about the patient and guiding what needed to be collected. In new cases, this related to past medical history, medications and available results of investigations. By contrast, what was reviewed during follow-up cases on the wards depended in part on how well the patient was already known to the trainee. Thinking tasks that were observable included forming a preliminary understanding of the patient’s contextual features (e.g., living situation, social supports, etc.), making connections between the patient’s medications and their past medical history, developing an understanding of the patient’s problem(s), and flagging gaps that exist within their own knowledge.

By combining knowledge of the presenting complaint, or known active issues, with the context provided by older notes, trainees could also, in part, plan what they needed to determine during their encounters. Junior trainees commonly wrote down notes on pertinent information collected before the patient encounter. They would then use this to guide what still needed to be collected or verified during the encounter. During new cases, for example:

*My preference is to write things out beforehand so that I have a thorough understanding of the patient’s backstory as much as possible and then I can complete details and verify details… just for efficiency’s sake as well as continuity’s sake, and safety’s sake, I write it out first, verify with the patient and then fill in details as needed.* (Table 1-1B)

Field interview 13, IM576 (PGY1).

Trainees across all levels employed a similar approach, but there were noticeable differences across levels of experience. Less experienced trainees were less selective in their collection and analysis of information, often electing to read and record all available information:

*He then opens a discharge summary from a few years back. Noting the comments on Chronic Obstructive Pulmonary Disorder (COPD), he comments that he needs to read more... writes TIA [transient ischemic attack] on his chart paper, below COPD. He then adds the ulcer below TIA, re-ordering the issues from the discharge summary…* (Table 1-1C)

Field visit 5, CC977 (Medical student).

Trainees with more experience also reviewed pre-existing notes. However, they were more selective in what they considered, trusted and wrote down:

*If it’s [medication list] more than a month old, I feel like things could have changed. And even then, I don’t try to rely on it too much, because there can always be errors in dictation…* (Table 1-1E)

Field interview 26, IM738 (PGY2).

In addition to accurately capturing the information, reviewing and writing out the past medical history and medication list for a new patient, especially for more junior trainees, could be used to help them to link the medications with chronic active problems:

*… Now that I’m more familiar with the medications, in my head, I can match them with their indication and see what’s what… I used to number them, based on the past medical history.* (Table 1-2B)

Field interview 23, IM133 (PGY2).

The review and writing out of the medications could also be used to identify active issues or potential causes for active issues:

*He reads over the list of meds on the summary and begins to write them out… He gets seven of the nine down and then stops to wonder aloud, “Why is she on [this medication]?” He then flips over to a service consult note and begins to read through it…* (Table 1-2A)

Field visit 9, IM939 (PGY1).

The pre-review of existing clinical investigations could be used to help identify active issues or potential causes of active issues. It could also be used to guide early ordering of new investigations in the emergency department or on the wards:

*…She highlights one line and appears to read it more closely. She then grabs the phone and calls the core lab, requesting that they add additional bloodwork for the sample they have drawn from this patient.* (Table 3-2C)

Field visit 22, IM289 (PGY3)

*… She pauses as she sees that the urine electrolytes and osmolality have not been checked yet and comments that these need to be done. She adds an order for the required tests.* (Table 3-2D)

Field visit 24, IM425 (PGY2).

While the most experienced trainees that we observed wrote less down during pre-review, they were still observed to spend considerable time reviewing patient records before encounters:

*He spends about 2 minutes reading this note before exiting and opening an older progress note... Throughout this entire process, he has not written anything down….* (Table 1-1F)

Field visit 18, IM865 (PGY3).

### After the patient encounter

After talking to and examining the patient, trainees shifted their focus to a different set of tasks. While trainees now needed to document a complete note with all of its traditional sections, the primary focus was on making sense of—or for follow-up cases updating—the problem list and formulating an assessment and plan. After the patient encounter, synthesizing the data and formulating a problem list was a key focus. The first step in this process was organizing all patient information, including details from the history, physical exam and investigations. For junior trainees, writing was consistently used to assist with this, at times, lengthy and thinking-intensive process:

*Sitting down at the computer station, CC977 prepares to work through the information he has collected… He begins to reflect aloud on what he wrote, verbally walking through some of the findings… After reflecting on it, he explains to me that he likes to talk it over to see if he has it all … Upon reflection, we return to the room to ask another question and CC977 immediately adds the patient’s answer into the chart. We leave the room and CC977 begins to write down some more exam findings in the appropriate section of his note.* (Table 2-1A)

Field visit 5, CC977 (Medical student).

With the most junior trainees, documentation sometimes proceeded in a more formulaic way, with a focus on writing to fill in the expected values of a section before making sense of what the written information itself meant:

*She then begins to add bloodwork values, using the computer that is still logged into Power-chart... She then jumps back to the A/P section and adds M [for metabolic], writing down notes on the presence of an electrolyte disturbance. She pauses and then adds a note on the need to replace the electrolytes. She then paused for a moment to look at the electrolyte values and turned to ask the SMR about what she was seeing.* (Table 2-1B)

Field visit 9, CC100 (Medical Student).

Synthesis appeared to evolve with increasing experience. More senior residents were less tied to writing all details prior to synthesizing. In part, this was an evolving necessity as Senior Medical Residents [SMR] acknowledged needing to be more efficient given an increased workload:

*So as a PGY1 I used to do that. I would pretty much have a fully handwritten note. But now that I’m PGY2, especially as the SMR, it’s too busy. I can’t take the time to write something down... I feel pretty comfortable… I know the kind of checkboxes that I have to hit…* (Table 2-1C)

Field interview 26, IM738 (PGY2).

The development of an assessment and plan tended to follow the synthesis process described above and involved taking time for reflection:

*He then adds the heading for assessment and plan. He writes a summary of the patient across the top of this section… He adds 1) Weakness, then pauses and comments he is not sure what the cause is. The patient had described what she thought was the problem [Coumadin prescription] but he cannot make himself believe that’s it. He then returns to his note and adds two queries after the issue* [*similar process for 5 more issues*]. *He skims through what he wrote. Turning to the computer, he opens the labs page and reads through what has been done. He debates out loud whether he should do more bloodwork. He then flips over to the orders page and looks at the pending queue and makes note of some of the tests related to earlier issues. He turns back to his page and reads it over.* (Table 2-2A)

Field visit 10, IM939 (PGY1).

Regardless of context—follow-up on the wards or new patient in the emergency department—for junior trainees, the effort expended during composing appeared to influence the ensuing teaching and guidance received around a case. Those who invested less time and effort developing their ideas before presenting and those who felt pressure (usually internal vs. external from the SMR) to prepare quickly appeared to have less meaningful dialogue; instead, they would frequently be told what to think or do:

*As she writes out this issue, CC100 is just writing down what the SMR is telling her...* (Table 2-2B)

Field note 9, CC100 (Medical student).

For a new patient, in addition to case review, another observed activity that reflected the effectiveness of early composing practices was dictation. For medical students and some junior residents, their approach to dictation was to write notes by hand and then read their notes as a script during dictation. This was seen as advantageous because it helped organize their thoughts and ensure they didn’t miss pertinent points:

*CC681 reels off the identification information very quickly, with no issues or pauses as he talks. He then begins to read over his notes as he continues into the dictation.* (Table 2-2C)

Field visit 17, CC681 (Medical student).

Over time, however, residents began to reduce the level of detail in their handwritten pre-dictation admission notes. Instead, they used them to map out the pertinent features of a case and organize their thoughts:

*Because you get it within two hours of dictating your note, it’s mostly just for myself to organize my thoughts... So, I write down the pertinent negatives… But other things that are less pertinent or other negatives you just go through, usually just kind of review of systems things, I don’t really write them… I write down the most important negatives and then the positives…* (Table 2-2E)

Field interview 10, IM939 (PGY1).

Throughout residency, trainees described a gradual progression in which they became able to handle thinking through their cases with less reliance on writing. This was true for routine cases and when there was readily available information in the electronic health record (Table 2-2F).

However, writing was still used to handle more complex patients:

*Previously speaking, I would say I probably would fully write a note and then have my full investigations and more importantly the assessment and plan... If it’s a complicated case, then sometimes I’ll still write down what I’m thinking. But most of the time nowadays I don’t feel like I need to do that… I guess this is more of a case where say, at the beginning of PGY1 year I would do it 100% of the time, where I would write down all of my notes and then dictate. And towards the end, I was doing it 20-30% of the time. And then by the time I got to PGY3 I was basically doing like 1-2% of the time.* (Table 2-2G)

Field interview 19, IM865 (PGY3).

### Identifying gaps

Before and after the patient encounter, trainees also identified gaps in personal knowledge and data collection. Examples of such gaps might include not recognizing a medication or being unfamiliar with a particular medical problem:

*… Moving on to the medications now, IM939 googles the first med that he sees listed [in the corresponding section of the admissions note] to see what it is.* (Table 7-1-A)

Field visit 10, IM939 (PGY1)

*I’ll see in their past medical history something that I’m not familiar with and that’ll be a trigger for me to look at Up-To-Date on what is this, what do I need to look for…* (Table 3-1B)

Field interview 5, CC977 (Medical student).

In the case of data collection gaps, there were multiple points in the composing process which facilitated the recognition of these; most junior trainees were frequently observed to recall additional pieces of information that were required while writing a clinical note:

*As she is reading the chart and documenting her findings, she realizes that she has not done a full neurological exam and will need to go back to do one on the patient.* (Table 3-2A)

Field visit 9, CC100 (Medical student).

Data collection gaps for junior trainees were also commonly noted during the process of case review:

*Scanning through the chart, IM605 interrupts the presentation to comment that there is a note in the chart about a heart condition. CC594 does not appear to have previously read about this condition… IM605 then tells CC594 to go back and see the patient again, to clarify a few of the things the presentation has shown he did not obtain the first time.* (Table 3-2B)

Field visit 14, IM605 (PGY3), CC594 (Medical student).

## Discussion

To broaden the debate on the educational value of documentation, building on work from the field of rhetorical genre studies, we explored the relationship between writing and thinking in junior and senior medical trainees. In addition to confirming and contextualizing the Paré and Smart model of composing practices (Paré & Smart, 1994), our study demonstrates the important and complicated developmental relationship between writing and thinking in the clinical setting. As some have problematized the educational value of documentation and the proportion of time it occupies, (Dresselhaus et al., 1998; Petrany, 2013) our findings also have implications for three inter-related discussions in the field: scut work, medical scribes in residency training, and electronic documentation.

Our key study finding is that for clinical trainees and educators, the term clinical documentation may represent a problematic misnomer serving to direct our attention to only one of its purposes—documentation requirements. We would argue that the better term is composing, which captures the more complex interaction observed between writing and thinking. While the most senior residents consistently appeared to be able to make do with more minimalist writing during the composing process, this was only possible for patients where existing clinical notes on the electronic health record provided most of the required contextual data (i.e., past medical history and medication list) and, when the patient cases represented relatively straightforward problems from that resident’s perspective. For all other trainees, writing played an essential role in supporting their thinking in their patient encounters.

Our findings also confirm Paré and Smart’s model of composition and draw attention to several contextual features that may help the struggling trainee and those new to the clinical environment. First, information gathering and analysis are an iterative process that begins before seeing the patient and continues through to after the initial encounter is complete—some trainees need to return to the bedside more than once to gather and confirm data. Second, junior trainees require considerable time post-encounter to write and think as they make sense of the data. Finally, shortchanging the thinking process has consequences; whether it occurs through early prompted review or through self-initiated early review, skipping the key thinking steps led to very different case reviews; ones characterized by minimal discussion and teaching and more telling of what to write and what to do.

While many of the observed junior trainees used a focused approach—an approach guided by the patient’s identified issues—to guide their writing and thinking prior to seeing a patient and following an encounter, not all trainees recognized or meaningfully engaged in the associated thinking tasks. Rather, similar to findings from Cadieux et al. (Cadieux & Goldszmidt, 2017), some medical students and junior residents appeared to not recognize the associated thinking required and instead approached composing as a formulaic documentation task predicated on filling in information into key sections of the note.

Both our findings and prior research suggest that learning composing occurs through trial and error, with inconsistent effort to directly tell trainees where their priorities should be and why (DeLeon et al., 2018; Rowlands et al., 2016). While prior efforts have shown that documentation quality can be improved with feedback (DeLeon et al., 2018; Opila, 1997; Rowlands et al., 2016), we would suggest that using our findings to teach trainees about composing might help them to not only improve documentation quality but also to better understand where they should focus their attention.

From an RGT perspective, there is an important and complicated developmental relationship between composing, thinking and attention that argues for the importance of composing beyond its direct influence that we studied (Bawarshi, 2001; Bazerman, 2009). During composing, according to Charles Bazerman, a leading RGT theorist: “genres identify a problem space for the developing writer to work in [e.g., focusing attention on the tasks associated with admitting a patient to hospital] as well as provide the form of the solution the writer seeks and particular tools useful in the solution. Taking up the challenge of a genre casts you into the problem space and the typified structures and practices of the genre provide the means of solution [e.g., following the admission genre can act as a scaffold to support thinking] (page 291)” (Bazerman, 2009). While not explicitly addressed in our study, according to RGT, composing’s developmental influence extends beyond the task itself. Over time, composing with the genres of the profession supports trainee socialization and identity development: “When communicants use genres, they are interpreting and enacting the social motives (embedded rhetorically within it) that sustain an environment and make it meaningful, and so are becoming socialized into producing not only certain kinds of texts, but also certain kinds of contexts, practices, and identities—ways of being and acting in the world, socially and rhetorically (page 78)”(Bawarshi, 2001). Therefore, the more a trainee grapples with the development of expertise in the profession and how to effectively communicate it through the genres of the profession, the more they begin to think like and enact the roles of the profession.

Some have considered documentation to be a “scut” like task in medicine, with little educational value relative to the proportion of time it occupies (Dresselhaus et al., 1998; Petrany, 2013). This perspective has also led some to suggest the use of medical scribes to reduce trainee time spent on “low yield” documentation activities (van Schaik, Reeves, and Headrick 2019). Our findings argue against this stance, and we would caution against the use of medical scribes in all but the most advanced trainees. While it is true that, when done well, writing can be time-consuming, it is necessary developmental work and should not be dismissed. Over time, senior trainees appeared to develop the ability to abbreviate the written component of composing. However, these trainees commented that writing was essential in their personal development and that they could not perform at their current levels of proficiency without first learning how to do it through writing.

While our study did not take place in a context where trainees entered text directly into the electronic health record—they dictated their clinical notes—our findings may have important implications for contexts where they do. Others have written about concerns related to cut and paste and the propagation of errors (Koppel, 2014; Siegler & Adelman, 2009), the use of templates frequently conveying false negatives (J. E. Siegler, Patel, and Dine 2015), and templates and checkbox menus interfering with trainees independently learning which items they need to document (Mintz et al., 2009). What our findings add are concerns related to cut and paste or pre-populated fields and the ways they might interfere with the necessary thinking that junior trainees may not recognize that they need when seeing patients. While some institutions have policies prohibiting medical students from copying information due to the risk of inaccuracies in data collection (Weis et al., 2014), we would argue that an equal concern is its impacts on learning and the provision of care.

It is important to consider our findings within the limitations of our study. Our results were obtained exclusively from Internal Medicine and it is unclear how these findings may transfer to other specialties. Additionally, while we can speculate on implications for contexts where direct electronic health record documentation takes place, unknown are the possible alternative strategies that trainees might develop for engaging in the necessary thinking when writing certain sections of the document is unnecessary. Finally, our study took place in a context where documentation was very much directed towards patient care. However, we recognize that regulatory and billing requirements in some countries may lead to trainees being involved in other forms of documentation that may not be as clearly linked to clinical care. In these contexts, it may very well be appropriate to consider strategies for reducing trainee involvement in certain forms of documentation to enhance the time they can spend on more meaningful activities like composing.

## Conclusion

Our findings demonstrate that the processes of writing and thinking are tightly linked in medical trainees through the process of composing and that the process of writing during composing is an essential developmental step for junior trainees. While some have described documentation as a ‘scut’ like activity and have proposed strategies for reducing trainee involvement in it, we would argue that writing is thinking and learning and that it can be time well spent.

## Appendix

See Tables 1, 2 and 3.

**Table 1:** Before the patient encounter

1	Preliminary Understanding of Contextual Features	
A	Using notes to prepare a set of questions	*Based on the active issues, I’ll kind of go in with a pre-set of questions I want to ask them about. And then, you kind of, well I kind of just… Based on how the interview’s going, you can just ask around whatever they bring up, ask around that. Because…of course, you have your own agenda in a sense, you have essential questions that you do not want to miss* Field Interview 2, CC365 (Medical student)
B	Writing information first to identify, then verifying with patients	*My preference is to write things out beforehand, so that I have a thorough understanding of the patient’s backstory as much as possible and then I can complete details and verify details, for efficiencies sake, with the patient… So, an example would be the past medical history. That’s not something that I necessarily need to reinvent with the patient then and there. Nor often do some of our more elderly patients especially, understand the full spectrum of medical issues that they have. So, I would say it’s much more thorough and much safer for me to rely on the history from a previous medical professional, write that down and then verify as much as I can with the patient. If they can’t remember it, well then so be it. It will still say on my record if it’s been past of past, trusted medical records. But yeah, just for efficiencies sake as well as continuities sake, and safeties sake, I write it out first, verify with the patient and then fill in details as needed* Field interview 13, IM576 (PGY1)
C	The formulaic approach to writing taken by Junior trainees	*He then opens a discharge summary from a few years back. Noting the comments on Chronic Obstructive Pulmonary disorder (COPD), he comments that he needs to read more. He doesn’t write anything as he had already added COPD to his note. Reading further, he sees a comment on a perforated duodenal ulcer, then immediately reads below it and sees mention of a Transient Ischemic Attack (TIA). He says this is important and writes TIA on his chart paper, below COPD. He then adds the ulcer below TIA, re-ordering the issues from the discharge summary. CC977 says that reading these notes before going to see the patient is key. He stops writing and returns to reading, saying that he wants to find more recent respiratory test values. He opens another note but is unable to find the values he is looking for. However, he notes a record of smoking pack-years and immediately writes this on the chart paper, remarking that it is a large number. He then stops writing and moves to another note on the computer. He reads about an excision of a melanoma, says that it is important and again writes on the chart paper, under his PMHx heading. He stops writing and then moves to another note, noting a second melanoma excision. I did not see him make any notes of this on his paper. CC977 moves to a more recent note and sees hypertension mentioned in the PMHx section of that note. This section also mentions a hip replacement. CC977 remarks that none of the other notes have mentioned these and adds both to the PMHx he is creating. After this, CC977 says we’re ready to go and see the patient* Field visit 5, CC977 (Medical student)
D	Selective use of source reading materials	*IM133 allows the CC to cover the patient’s story and then asks him for a medication list. The CC has a list of medications which he obtained from an older note. IM133 tells him to go back and ask the patient for a list, as the note was a little old and is not the most reliable for an accurate list. She suggests using the ER progress notes, as they will often obtain a medication list early in the course of treating a patient… Having read the same things as CC, IM133 knows that he got his original list of medications from a discharge summary done in March. She mentions that this is too old to be relied upon as up to date. IM133 mentions ClinicalConnect to CC now as a resource that can be used and loads it to show him the pharmacy and home medication tabs* Field visit 23, IM133 (PGY2)
E	Sources for reliable medication lists	*Maybe I won’t be able to answer this directly, but the way that I approach that is that if they’re above the age of 65 I go on ClinicalConnect and try to obtain their medications, because they’ll be there. And then if that’s not the case then I go to the patient. And a lot of times actually our patients come in with blister packs, so you can just look on that. Or a list of their medications. So, I usually try to do that. And then, to try to answer your question… I don’t know, I guess if… If it’s more than a month old, I feel like things could have changed. And even then, I don’t try to rely on it too much, because there can always be errors in dictation. So, I usually don’t try to rely on previous notes for medications, that’s how I approach that problem* Field interview 26, IM738 (PGY2)
F	Selectively seeking information without rewriting of information	*The previous SMR now leaves and IM865 re-opens one of the patients in his list, who has not yet been seen. He goes to her investigation results page and spends about 40 s reading the lab values. He then flips over to the notes section and opens a recent discharge summary… He spends about 2 min reading this note before exiting and opening an older progress note. He scrolls down and skims a few lines, spending perhaps 5 s total on this note. He then picks another progress note and opens it, spending about 30 s scrolling through it. He then returns to the investigations page and scrolls along the entire thing, reading over values. Throughout this entire process, he has not written anything down… When we arrive, IM865 grabs the ER chart and glances through it quickly. There is an ECG tracing, which he takes out and looks over. He then finds an envelope with a report that appears to be from EMS. He reads this report for about 30 s. There is also a transfer discharge summary, which he skims through quickly, flipping between the pages. We then go in to interview the patient… I note that IM865 is very purposeful and direct in his questions* Field visit 18, IM865 (PGY3)
2	Connecting PMHx and Medications	
A	Seeking clarification on recorded medication purposes	*He reads over the list of meds on the summary and begins to write them out, again flipping his gaze between the page and the computer screen. He gets seven of the nine down and then stops to wonder out loud “Why is she on Ezetimibe?” He then flips over to a service consult note and begins to read through it, seeking an answer. After reading for a moment, he comments that referral back to this doctor is needed to sort some issues out* Field visit 9, IM939 (PGY1)
B	Matching medications to past medical history	*So, medications are organized alphabetically and not by their indication. But, now that I’m more familiar with the medications, in my head I can match them with their indication and see what’s what…I used to number them, based on the past medical history* Field interview 23, IM133 (PGY2)
C	Identifying patient medications	*After she gets off the phone, IM289 returns the screen to ClinicalConnect. She grabs her pen and begins to write a list of medications down on her growing note, glancing back and forth between the computer screen and paper as she writes. After copying about six things down, she exits ClinicalConnect* Field visit 22, IM289 [PGY3]
D	Writing to gather thoughts before dictation	*Earlier on I was writing a handwritten note. First it was sort of helping me to gather my thoughts and then formulate what I want to say. And then edit that note and then use it as dictation, for more reading off a template. But I think right now I don’t really need my notes for that purpose… But I do write the note because of the documentation purposes* Field interview 22, IM289

**Table 2:** After the patient encounter

1	Synthesizing the data and formulating a problem list	
A	Writing of preliminary note	*Sitting down at the computer station, CC977 prepares to work through the information he has collected… He begins to reflect out loud on what he wrote, verbally walking through some of the findings. He begins to add some observations to his note, such as “rolling around, can usually ambulate”. He overhears a nurse in the background mention Lasix for the patient, so he pauses his writing and pulls up the medication records in the computer to see what was given. As he reads the medications on the computer, he begins to add more details to his meds section on the note. He then flips to the labs page and examines the blood gases, reading each value out loud and deciding if it is good or bad. He points out some connections between the values, noting the elevated bicarbonate and carbon dioxide with a normal pH as compensated respiratory acidosis. He then begins to write the values in the chart after he has commented on whether they are good or bad. He pauses to find the electrolytes and blood cell counts, and then writes them into the note. He opens a chest X-ray and comments on it, pointing at the screen to note abnormalities. He does not write anything about the X-ray into the chart… Returning to the patient’s chart, he opens an ECG and works through it, verbally talking over each aspect. He doesn’t write anything as he analyses the ECG. Next, he reads the vitals off the computer, again commenting out loud what he thinks of each. He copies the vitals down but does not write his thoughts on them down. He writes down the volume of oxygen and the Venturi mask flow rate. As he does so, he comments that it’s “pretty high”. He stops to think for a moment, then writes “X-ray” onto his note because it was abnormal. He then pauses again and walks back through the ECG results verbally. After reflecting on it, he explains to me that he likes to talk it over to see if he has it all. At this time, CC977 stops writing again and thinks things over. He reads back through the chart he got from the nurse and then adds a note to his own chart about POCUS for DVT. As he does this, he wonders out loud if he should do any more work up for DVT. Upon reflection, we return to the room to ask another question and CC977 immediately adds the patient’s answer into the chart. We leave the room and CC977 begins to write down some more exam findings in the appropriate section of his note* Field visit 5, CC977 (Medical student)
B	Formulaic Writing	*She then begins to add bloodwork values, using the computer that is still logged into Power-chart. At the same time, she is listening to the SMR continue to explain the significance of the exam findings. She jumps ahead to write some more in the A/P section as she listens. She then returns to the objective section to add in the electrolyte values, reading off of the computer, still listening to the SMR. She then jumps back to the A/P section and add M [for metabolic], writing down notes on the presence of an electrolyte disturbance. She pauses then adds a note on the need to replace the electrolytes. She then paused for a moment to look at the electrolyte values and turned to ask the SMR about what she was seeing* Field Visit 9, CC100 (Medical student)
C	Writing to flag important points	*So as a PGY1 I used to do that. I would pretty much have a fully handwritten note. But now that PGY2, especially as the SMR, it’s just too busy. I can’t take the time to write something down. And… I feel pretty comfortable in my schemata in my brain, where I know the kind of checkboxes that I have to hit. And obviously writing would be more foolproof in terms of errors, but I usually don’t have a full note. I will still have highlights, like if a patient, for example, had a recent investigation done, I’ll write that down to make sure it’s highlighted in my brain. Yeah, I use notes mostly as like a flag almost now* Field interview 26, IM738 (PGY2)
2	Developing an Assessment and Plan	
A	Development of an assessment and plan through writing	*He then adds the heading for assessment and plan. He writes a summary of the patient across the top of this section, then stops for a few seconds. He adds 1) Weakness, then pauses and comments he is not sure what the cause it. The patient had described what she thought was the problem [Coumadin rx] but he cannot make himself believe that’s it. He then returns to his note and adds two queries after the issue… He reads over his HPI section and drugs, wondering out loud what he can hold. He seems to find something that he can hold safely after a few seconds. He then pauses and adds 2) A/C [anticoagulation], pauses for a second and then makes a note after this about potentially changing over to a DOAC. He stops for a second and then adds 3) AKI… IM939 then returns to his note, writing 4). He then hesitates and talks out loud for a minute. He has not seen much to indicate the patient needs diuresis, but he feels that she does. Since cardio also felt the same, he decides that they will give her some Lasix. He writes out the issue for number four, commenting next to it on the cardio view and the plan. He then adds 5) Abdo and makes a note on the X-rays he’s ordered. He then adds 6), stops and flips to the front page of his note. He reads over what he has and then flips back to the backside. He adds a note near the top of the page, then flips back to the front and skims through what he wrote. Turning to the computer, he opens the labs page and reads through what has been done. He debates out loud whether he should do more bloodwork. He then flips over to the orders page and looks at the pending queue and makes note of some of the tests that are for earlier issues. He turns back to his page and reads it over. He comments that he done with the note now, but almost immediately adds one more issue on the A/P and notes the steps being taken to control it* Field visit 10, IM939 (PGY1)
B	Lack of preparation before case review	*Moving on, CC100 begins to consider the issue of the AKI and lithium toxicity. As she writes out this issue, CC100 is basically just writing down what the SMR is telling her… She writes out that there’s an X-ray being done, then pauses to wonder why it is being done. The SMR explains the reason and then CC100 asks about the normal steps in a diarrhea case, working through them with the SMR* Field note 9, CC100 (Medical student)
C	Dictating from a script	*CC681 reels off the identification information very quickly, with no issues or pauses as he talks. He then begins to read over his notes as he continues into the dictation* Field visit 17, CC681 (Medical student)
D	Dictating from a script	*As a PGY1 and a med student, I would essentially have a full note done, like a full consult note written. And then when it came time to dictate, I would literally just say what I had written out loud… Like, every med student and PGY1 should be doing that. Cause it’s easy to miss stuff and you need to have that organization in your brain. And the only way you’re going to develop it is through repetition as a junior* Field interview 26, IM738 (PGY2)
E	Shift to more selective writing	*And then, cause we dictate out notes and you hit the STAT, so you get them within two hours of dictating your note. So, because you get it within two hours of dictating your note, it’s mostly just for myself to organize my thoughts. Then I dictate it and it’s nice and neat, I reference that instead of trying to read my writing. So, this is mostly just for my own personal thoughts… So, I write down the pertinent negatives. So, the things that are relative, relevant to the case. Like… pertinent things. But other things that are less pertinent or other negatives you just go through, usually just kind of review of systems things, I don’t really write them or else it’d take forever to write down. So, I write down the most important negatives, and then the positives, which there were very few of them for her. And I go from there* Field interview 10, IM939 (PGY1)
F	Internalized formulation for dictating at the senior level	*Earlier on I was writing a handwritten note. First it was sort of helping me to gather my thoughts and then formulate what I want to say. And then edit that note and then use it as dictation, for more reading off a template. But I think right now I don’t really need my notes for that purpose… Because most of the past medical history and medications required are already in the computer. So, I can just read off. And in terms of the history of presenting illness and the exam findings, and then what I want to say in terms of the assessment and plan, that formulation is in my head and I can just say it on the dictation. So, I don’t need the note to help remind me* Field interview 22, IM289 (PGY3)
G	Progression to dictation without prior writing	*Previously speaking, I would say I probably would fully write a note and then have my full investigations and more importantly the assessment and plan. I would essentially direct that. And then once I was confident in my plan, then I would dictate the whole thing. And basically, whether I was kind of dictating verbatim what I wrote down or adjusting things to add in further details, that’s what I would do… If it’s a complicated case, then sometimes I’ll still write down what I’m thinking. But most of the time nowadays I don’t feel like I need to do that… I would say towards the end of R1 year and the beginning of R2 year is probably when that transition happened. And again, I guess this is more of a case where say, at the beginning of R1 year I would do it 100% of the time, where I would write down all of my notes and then dictate. And towards the end, I was doing it 20–30% of the time. And then by the time I got to R3 I was basically doing like 1–2% of the time* Field interview 19, IM865 (PGY3)

**Table 3:** Both before and after the patient encounter

1	Knowledge Gaps	
A	Recognizing and correcting medication related knowledge gap	*After the presentation, he turns to the computer and pulls up a recent admission note from the file. He reads through the admissions note, scanning and highlighting blocks of text as he goes…Moving on to the medications now, IM939 googles the first med that he sees listed [in the corresponding section of the admissions note] to see what it is* Field visit 10, IM939 (PGY1)
B	Identification of knowledge gaps through reviewing prior clinical note	*Probably really complex presentations. Really rare stuff or stuff I don’t have experience with. Those would be triggers… You know, I’ll see in their past medical history something that I’m not familiar with and that’ll be a trigger for me to look at Up-To-Date on what is this, what do I need to look for… So basically things that are different where I’m looking for change, and then things I don’t understand fully will be a trigger for me to look either back in the chart for some explanation or into my own, like… Up-To-Date or some other resource* Field Interview 5, CC977
2	Data Collection Gaps	
A	Recognizing data collection gap while documenting findings	*Moving back to the ER progress notes, CC100 reads through the chart and the patient’s ECG. As she is reading the chart, she realizes that she has not done a full neurological exam and will need to go back to do one on the patient* Field visit 9, CC100
B	Recognizing data collection gap during case review	*IM605 sees one [a note] and enters it, interrupting the presentation to comment that there is a note here about a heart condition. CC594 does not appear to have previously read about this condition; he adds a note to his paper as IM605 mentions it…IM605 tells CC594 that he should look back and find an older ECG, so that he can contrast the findings on the patient’s ECG with the baseline…IM605 then tells CC594 to go back and see the patient again, to clarify a few of the things the presentation has shown he did not obtain the first time* Field visit 14, IM605 (PGY3), CC594 (Medical student)
C	Recognizing data collection gap during analysis	*IM289 then exits the note on the computer and clicks over to the investigation results section, glancing through the results quickly. She highlights one line and appears to read it more closely. She then grabs the phone and calls the core lab, requesting that they add additional bloodwork for the sample they have drawn from this patient* Field visit 22, IM289 (PGY3)
D	Recognizing data collection gap during analysis	*IM425 stays in this patient’s file for the moment. She returns to the investigation results page and looks through the results. She pauses as she sees that the urine electrolytes and osmolality have not been checked yet and comments that these need to be done. She adds an order for the required tests* Field visit 24, IM425 (PGY2)

## Declarations

### Previous presentations

Canadian Conference on Medical Education (Niagara Falls, Canada, April 14^th^, 2019), Western University Department of Medicine Research Day (London, Canada, May 10^th^, 2019), Centre for Education Research and Innovation Symposium (London, Canada, October 2^nd^, 2019).
